# Enhancement of phycocyanobilin biosynthesis in *Escherichia coli* by strengthening the supply of precursor and artificially self-assembly complex

**DOI:** 10.1016/j.synbio.2023.02.005

**Published:** 2023-02-28

**Authors:** Yuqi Wang, Ning Li, Xiaoyu Shan, Xinrui Zhao, Yang Sun, Jingwen Zhou

**Affiliations:** a Key Laboratory of Straw Comprehensive Utilization and Black Soil Conservation, Ministry of Education, College of Life Sciences, Jilin Agricultural University, Changchun, 130118, China; b Key Laboratory of Industrial Biotechnology, Ministry of Education and School of Biotechnology, Jiangnan University, 1800 Lihu Road, Wuxi, Jiangsu, 214122, China; c Science Center for Future Foods, Jiangnan University, 1800 Lihu Road, Wuxi, Jiangsu, 214122, China; d Jiangsu Provisional Research Center for Bioactive Product Processing Technology, Jiangnan University, 1800 Lihu Road, Wuxi, Jiangsu, 214122, China

**Keywords:** Phycocyanobilin, Heme, Metabolic engineering, *Escherichia coli*

## Abstract

Phycocyanobilin (PCB) is widely used in healthcare, food processing, and cosmetics. *Escherichia coli* is the common engineered bacterium used to produce PCB. However, it still suffers from low production level, precursor deficiency, and low catalytic efficiency. In this study, a highly efficient PCB-producing strain was created. First, chassis strains and enzyme sources were screened, and copy numbers were optimized, affording a PCB titer of 9.1 mg/L. Most importantly, the rate-limiting steps of the PCB biosynthetic pathway were determined, and the supply of precursors necessary for PCB synthesis was increased from endogenous sources, affording a titer of 21.4 mg/L. Then, the key enzymes for PCB synthesis, HO1 and PcyA, were assembled into a multi-enzyme complex using the short peptide tag RIAD-RIDD, and 23.5 mg/L of PCB was obtained. Finally, the basic conditions for PCB fermentation were initially determined in 250 mL shake flasks and a 5-L bioreactor to obtain higher titers of PCB. The final titer of PCB reached 147.0 mg/L, which is the highest reported titer of PCB so far. This research provided the foundation for the industrial production of PCB and its derivatives.

## 1 Introduction

Phycocyanobilin (PCB) processing from algae is a natural blue pigment with a linear tetrapyrrole structure. PCB has a wide range of medical applications due to its antioxidant, anti-inflammatory, anti-cancer, and other properties [1]. It can be used in the treatment of Parkinson's disease (PD) and Alzheimer's disease (AD) and as a potential drug to reverse COVID-19-induced neurological damage [2]. In addition, PCB can be used as an important intermediate in materials science and was an important component of genetic manipulators of optical switches [3]. At present, PCB mainly comes from plant extraction and chemical synthesis. Plant extraction to obtain PCB is through methanol pyrolysis of algae, but the growth cycle of algae is long, the extraction rate is low, and the extraction process is complex [4,5]. The inhibition of regioselective and stereoselective functionalization restricts chemical synthesis [3,6]. Therefore, the search for more efficient methods of PCB synthesis has become an area of intense interest.

**Fig. 1:**
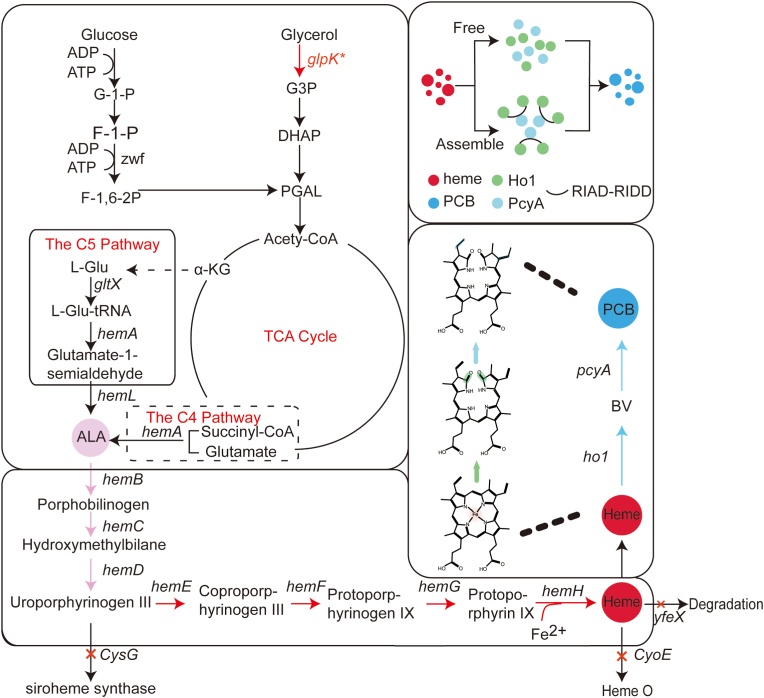
**Engineered metabolic pathway of phycocyanobilin in *E. coli****.* Overexpressed genes are highlighted in red; Heterologously expressed genes are highlighted in blue; Knocked-out genes are highlighted by a red cross.

In recent years, biosynthesis has become an effective way to increase the titer of some natural compounds that are widely used but limited in natural synthesis, such as 3,4-dihydroxyphenyl-l-alanine, phenylpropanoid acids, and 5-aminolevulinic acid (ALA) [[7], [8], [9]]. ALA synthesized via two metabolic pathways (C4 and C5) is the common precursor of all tetrapyrroles and plays an important role in plant growth regulation [10,11]. Researchers have obtained high titers of ALA in *E. coli*, *Corynebacterium glutamicum*, and *Saccharomyces cerevisiae* by screening multiple sources of *hemA* and *hemL* as well as constructing biosensors [[12], [13], [14], [15]]. ALA is converted to protoporphyrin IX by a series of porphyrin enzymes. Then, ferrochelatase embeds Fe^3+^ into protoporphyrin IX to form heme [11,16]. After heme formation, HO1 catalyzes the formation of biliverdin IXα (BV IXα) from heme, and BV IXα forms PCB in the presence of Phycocyanobilin: ferredoxin oxidoreductase (PcyA) (Fig. 1)*.* Therefore, based on the current study, it is possible to *de novo* synthesize PCB [[17], [18], [19], [20]].

As an engineered bacteria with well-studied genetic and metabolic information, *E. coli* has been widely utilized to synthesize a variety of substances [21,22]. Previously, researchers regulated the heme biosynthesis pathway and expressed *ho1* and *pcyA* genes to enhance PCB titer to 6.64 mg/L in *E. coli* [17]. Immediately, the titer of PCB was increased to 13 mg/L by optimizing the fermentation conditions [18]. More recently, by constructing a DNA scaffold to assemble *ho1* and *pcyA*, improving the cofactor cycle, adding the precursor ALA directly, and overexpressing *hemB* and *hemH* [19], the highest PCB titer (28.32 mg/L) was obtained in *E. coli*. However, the genes involved in the PCB synthesis pathway were different, and the titers of PCB were still low. In addition, the endogenous synthesis of heme, an important precursor for PCB synthesis, remains limited. Thus, the biosynthesis of PCB in *E. coli* has many issues that need to be solved urgently.

In this study, an efficient PCB-producing strain was constructed through metabolic engineering. First, to create optimal conditions for PCB synthesis, a better chassis strain was screened. Then, the PCB titer was further increased by integrated expression of key genes involved in precursor synthesis. Moreover, through the binding of the RIAD-RIDD short peptide tag to *ho1* and *pcyA* gene expression, the catalysis rate of heme to PCB was enhanced. In addition, several commonly used fermentation media in laboratories were compared, along with induction temperatures, and a fermentation temperature of 25 °C and GMD-Gly medium were selected. Finally, an expanded culture of PCB synthesis was performed in a 5-L bioreactor to verify the potential of the best-engineered strain for PCB production. We obtained 147.0 mg/L, the highest PCB titer among microbial synthesis reported to date.

## 2 Materials and methods

### 2.1 Chemical compounds and standards

High fidelity DNA polymerase Phanta® Max Master Mix and a FastPure® Gel DNA Extraction Mini Kit were purchased from Vazyme Biotech Co., Ltd (Nanjing, China). All chemical reagents not described above were purchased from Sangon Biotech Co., Ltd (Shanghai, China). All genes had been cloned in previous work [19]. Oligonucleotide primers were synthesized by Sangon Biotech Co., Ltd. (Shanghai, China). All other chemicals and reagents were purchased from Shanghai Sangon Bio-chemical Co., Ltd. (Shanghai, China). The standard of phycocyanobilin (PCB, >95%) was purchased from Frontier Specialty Chemicals [19].

TB medium: 10 g/L tryptone, 24 g/L yeast extract, 5 g/L glycerol, 2.31 g/L KH_2_PO_4_, 12.42 g/L K_2_HPO_4_.

FLB medium: 35 g/L glucose, 5 g/L (NH_4_)_2_SO_4_, 3 g/L KH_2_PO_4_, 3 g/L MgSO_4_⸱7H_2_O, 1 g/L NaCl, 1.5 g/L Trisodium citrate dihydrate, 0.015 g/L CaCl_2_⸱6H_2_O, 0.1125 g/L FeSO_4_⸱7H_2_O, 0.075 g/L vitamin B1, 4 g/L tryptone, 2 g/L yeast extract.

M9 medium: 0.2 g/L NH_4_Cl, 0.1 g/L NaCl, 0.6 g/L KH_2_PO_4_, 2.56 g/L Na_2_PO_4_⸱7H_2_O, 4 g/L glucose, 2.191 g/L CaCl_2_⸱6H_2_O, 2.46 g/L MgSO_4_⸱7H_2_O

GMD-Gly/GMD-Glu medium: 6 g/L K_2_HPO_4_, 16.4 g/L K_2_HPO_4_⸱3H_2_O, 1 g/L citric acid, 1 g/L MgSO_4_⸱7H_2_O, 10 g/L yeast extract, 30 g/L glycerol/glucose.

GMD-Gly fermentation medium: 6 g/L KH_2_PO_4_, 16.4 g/L K_2_HPO_4_⸱3H_2_O, 5 g/L (NH_4_)_2_SO_4_, 1 g/L citric acid, 1 g/L MgSO_4_⸱7H_2_O, 10 g/L yeast extract, 30 g/L glycerol, 10 g/L maltodextrin, 0.1 g/L vitamin B1, and 1 mL/L trace elements.

The trace elements: 100 g/L Fe (III) citrate, 18 g/L ZnCl_3_, 14.64 g/L MnSO_4_·H_2_O, 0.75 g/L CuSO_4_·5H_2_O, 2 g/L Na_2_MoO_4_·2H_2_O, 2 g/L CaCl_2_.2H_2_O, 3.0 g/L H_3_BO_3_, 2.5 g/L CoCl_2_.6H_2_O, 2.5 g/L NiSO_4_.6H_2_O, and 100 mL HCl.

### 2.2 Strains and culture conditions

*E. coli* JM109 was used for plasmid construction. *E. coli* strain St06, possessing heterologous genes for *de novo* production of PCB, was constructed in previous work [19]. All strains used in this study are listed in Table S1. *E. coli* were activated in Luria-Bertani (LB) medium at 37 °C. Ampicillin (50 mg/L), kanamycin (50 mg/L), chloramphenicol (25 mg/L), or spectinomycin (50 mg/L) was used to culture the *E. coli* strains as required.

### 2.3 Genetic operations

Molecular cloning and enzyme digestion techniques were used for plasmid construction. The plasmids and primers are listed in Tables S1 and S3. The CRISPR/Cas9 gene editing system was used to knock-out or integrate the genes of the strains’ genome [23]. The plasmids pCas9 and p-Target for the CRISPR/Cas9 gene-editing system were kindly provided by the Shanghai Institute of Plant Physiology and Ecology [23]. Refer to this document for details of the operation [24].

### 2.4 Cultivation in shake flashes and bioreactors

Shake flash conditions: The seed solution was incubated at 37 °C and 220 rpm for 10–12 h. The seed solution was then transferred to the fermentation medium at an inoculum level of 2%. When the OD_600_ was 0.6–0.8, the appropriate concentrations of isopropyl-β-d-thiogalactopyranoside (IPTG) were added to induce the synthesis of PCB at 25 °C. The concentration of the IPTG was 0.5 mM. According to plasmid requirements, appropriate concentrations of antibiotics were added, including ampicillin (50 mg/L), kanamycin (50 mg/L), chloramphenicol (25 mg/L), or streptomycin (50 mg/L).

Fed-batch fermentation conditions: The secondary seed solution was added to the 5-L bioreactor with 2.5-L of the fermentation medium. During the early stage, the temperature was controlled at 37 °C. The IPTG was added when there was a rebound in dissolved oxygen (DO), at which time the temperature was cooled to 25 °C. In the fermentation process, the pH was controlled at 7.0 by the automatic addition of 50% ammonia. The dissolved oxygen and speed were adjusted appropriately according to the situation.

### 2.5 Analytical method

The fermentation broth was prepared with an equal volume of methanol. The mixture was heated in a water bath at 50 °C for 1 h and then shaken for 5 min. After centrifugation, the supernatant was filtered through a 0.22 μm membrane. The concentration of PCB was analyzed by High Performance Liquid Chromatography (HPLC) with a C18 column (30 cm × 0.25 cm) at 25 °C. A wavelength of 380 nm and a flow rate of 0.8 mL/min were used [19].

## 3 Results

### 3.1 Screening of chassis strains and construction of PCB synthesis pathway

**Fig. 2:**
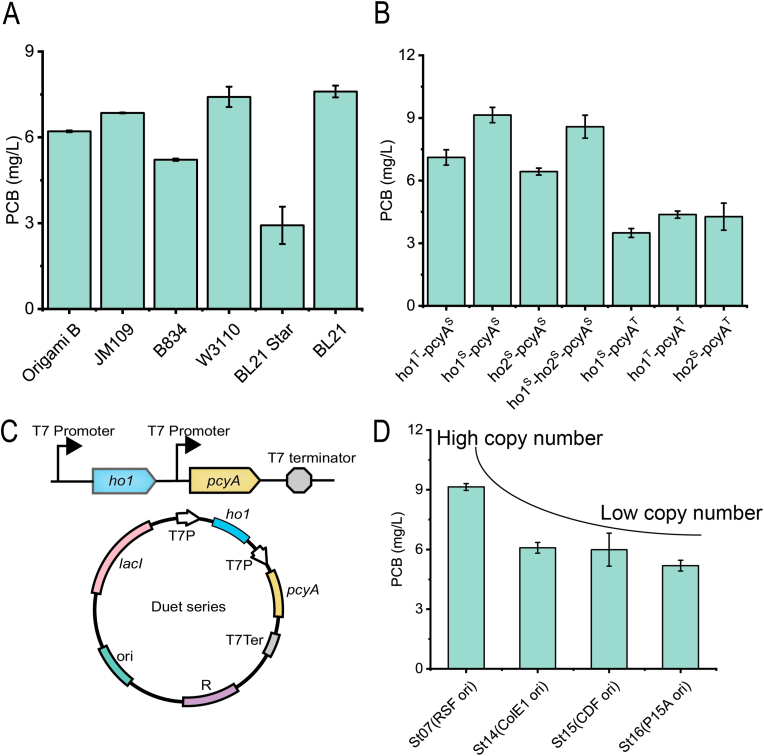
**Construction of the initial strain.** A: Effect of chassis strains on PCB synthesis; B: effect of different sources of enzymes on PCB synthesis; C: schematic diagram of Duet series plasmid construction; D: effect of copy number on PCB synthesis.

Previous studies suggested that using two independent T7 promoters to express *ho1* and *pcyA* was better for the production of PCB compared with both genes controlled by a T7 promoter [17]. To discover the most suitable *E. coli* strains for the synthesis of PCB, the strains JM109(DE3), B834(DE3), W3110(DE3), BL21 Star(DE3), Origami B(DE3), and BL21(DE3) were compared. The plasmid pRSFDuet-P_T7lac_-*ho1*^T^-P_T7lac_-*pcyA*^S^ was introduced into these strains, and the strain St06 was the best (Fig. 2A). *Ho1*^*S*^ and *ho2*^*S*^ are a homolog of the heme oxygenase gene, and both have comparable activities. *Ho1*^*T*^ and *pcyA*^*T*^ derived from *Thermosynechococcus elongatus*BP-1 were used instead of *ho1*^*S*^ and *pcyA*^*S*^ in *E. coli* BL21(DE3). To compare the ability of the two key enzymes to synthesize PCB, they were individually expressed or co-expressed in *E. coli* BL21(DE3) to obtain strains St07, St08, St09, St10, St11, St12, and St13. The strains St12 and St13 yielded 4.2 mg/L and 8.4 mg/L of PCB, respectively. The strains St10 and St11 produced 3.5 mg/L and 4.3 mg/L PCB, respectively. These titers were much lower than that of the strain St07 (Fig. 2C). The titer of the strain St07 was 9.1 mg/L. The results indicated that Ho1 and PcyA from PCC 6803 were relatively more suitable for PCB synthesis in *E. coli* (Ho1^S^ and PcyA^S^ derived from PCC6803 are abbreviated as Ho1 and PcyA).

Therefore, *ho1* and *pcyA* from PCC 6803 were selected for further study. On this basis, *ho1* and *pcyA*, which were derived from *Synechocystis* sp. PCC6803, were simultaneously cloned into other plasmids with different copy numbers to obtain pETDuet-*ho1*-*pcyA* (medium copy number), pCDFDuet-*ho1*-*pcyA* (medium copy number), and pACYCDuet-*ho1*-*pcyA* (low copy number) (Fig. 2B). Then, the constructed plasmids were introduced into *E. coli* BL21(DE3) to generate the strains St14, St15, and St16. However, the strain St07 (pRSFDuet-*ho1*-*pcyA*, high copy number) was still the highest PCB-producing strain. The titers of the strains St14, St15, and St16 were 6.0 mg/L, 5.9 mg/L, and 5.2 mg/L, respectively (Fig. 2D). The results showed that increasing copy numbers of *ho1* and *pcyA* genes slightly improved the production of PCB.

### 3.2 Enhanced precursor by integrated expression of key genes

**Fig. 3:**
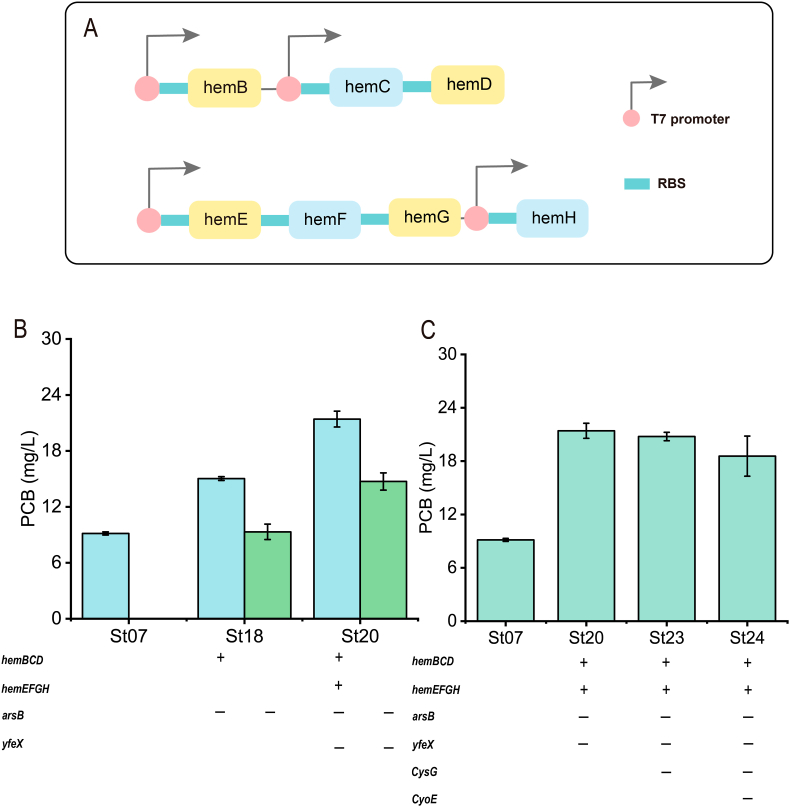
**Effect of genome integration on PCB synthesis**. A: Schematic diagram of the structure of the integrated heme gene; B: effect of integration precursors and knockout integration sites on PCB synthesis. Integration in blue, knockout in green; C: effect of knockout of *CysG* or *CyoE* on PCB synthesis. ‘+’ means gene integration, ‘-’ symbol means gene knockout. The blank means without genetic modification.

Heme synthesis was encoded by seven genes: *HemB*, *hemC*, *hemD*, *hemE*, *hemF*, *hemG*, and *hemH*. Heme was a precursor to PCB biosynthesis (Fig. 1). However, it only produced a trace titer of heme in wild-type *E. coli* [11]. Therefore, P_T7_-*hemBCD* was integrated into the *arsB* locus of the *E. coli* BL21(DE3) genome using the CRISPR/Cas9 gene editing system to increase the endogenous synthesis of heme. Then, the correctly sequenced strain St17 was obtained. The plasmid PW07 (pRSFDuet-*ho1*-*pcyA*) was introduced into the strain St17 to obtain the strain St18. It was found that the strain St18 increased the PCB of the initial strain St07 by 64% to 15.0 mg/L. Next, P_T7_-*hemEFGH* was integrated into the *yfeX* locus of the strain St17 to obtain the strain St19. The gene *yfeX* from *E. coli* had been reported to inhibit heme synthesis [25]. Similarly, the plasmid PW07 was introduced to obtain strain St20. Surprisingly, the PCB titer of the strain St20 reached 21.4 mg/L, which was 134% higher than the control strain St07. The result suggested that genomic integration of key genes of the precursor synthesis pathway was effective for increasing the titers of PCB (Fig. 3).

### 3.3 Knockout of competing pathways

To further improve the synthesis of endogenous heme, the genes *CysG* and *CyoE* on the genome of the strain St19 (*E. coli* BL21(DE3) Δ*arsB*:*hemBCD* Δ*yfeX*:*hemEFGH*) were knocked-out. The gene *CysG* can convert uroporphyrinogen III to the precursor to cobalamin and siroheme, while *CyoE* degrades heme into Heme O (Fig. 1) [26,27]. Therefore, the strains St21 and St22 were constructed. After introducing the plasmid PW07 into these two strains, the strains St23 and St24 were obtained, respectively. Unexpectedly, the PCB titers both of the two strains were reduced. The strain St23 produced 20.7 mg/L of PCB, while the strain St24 produced even less, at 18.5 mg/L. Although the gene *CysG* converts uroporphyrinogen III to the precursor to cobalamin and siroheme, this may result in a reduction in the synthesis of heme. However, siroheme still forms Fe-coporporphyrin III to synthesize heme, which is presumably one of the reasons for the reduction in PCB production [28]. As for *CyoE*, hemO is also considered a form of heme and was involved in other metabolic pathways, so knocking out *CyoE* would affect the balance of heme metabolism in cells. Therefore, the strain St19 was still chosen as the chassis strain for PCB synthesis.

### 3.4 Expression of Ho1 and PcyA by artificially self-assembly complex

**Fig. 4:**
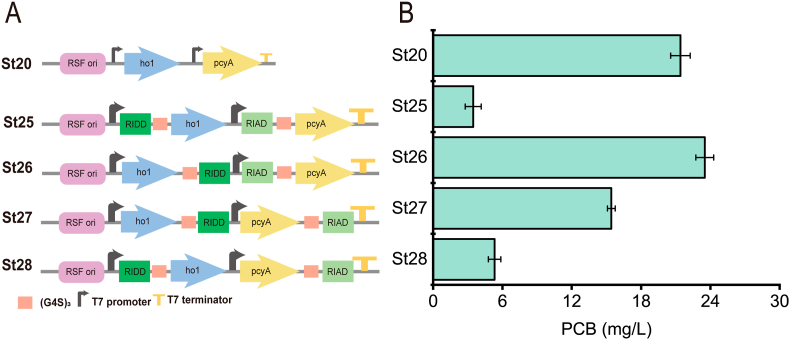
**Effect of different linkages between RIDD-RIAD and HO1-PcyA. A**: Structure of the artificially self-assembly complexs with different linkage positions; B: the PCB titer corresponding to Figure A.

After heme was synthesized in a high titer, to convert heme to PCB efficiently and further improve the catalytic efficiency of Ho1 and PcyA, four artificially self-assembly complexs were constructed (Fig. 4A). Here, this pair of self-assembled peptide tags was used to spatially associate the important pathway enzymes for PCB biosynthesis in recombinant *E. coli* cells to increase PCB productivity and yield [29]. The linker GGGGS was used to attach the pair of short tags RIAD-RIDD to the C- or N-termini of *ho1* and *pcyA* to compare the effects of different attachment positions on PCB synthesis. The strains St25, St26, St27, and St28 were obtained. The shake flask fermentation results showed in Fig. 4B that the artificially self-assembly complex system boosted PCB synthesis and effectively increased catalytic efficiency when the two short peptide tags were attached between the two enzymes. The titer of strain St26 was 23.5 mg/L and increased by 157% compared to strain St07.

### 3.5 Fed-batch fermentation in a 5-L bioreactor

**Fig. 5:**
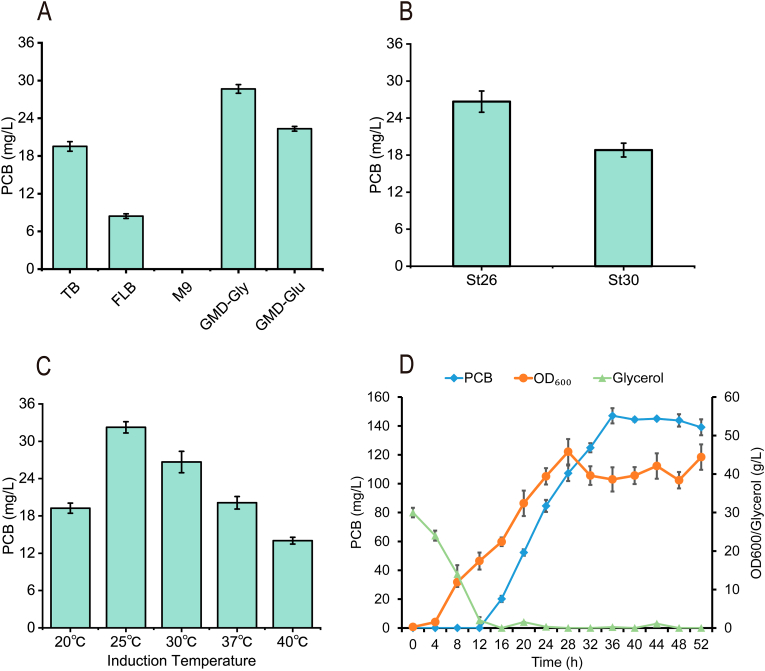
**Optimization of PCB production in 250 ml shake flasks and 5-L bioreactor. A**: Comparing the different components of the culture media; B: Mutations in the *glpK* gene; C: Contrast of different induction temperatures; D: Fed-batch fermentation by feeding with 500 g/L glycerol, 7.5 g/L MgSO_4_·7H_2_O, 2.5 g/L of (NH_4_)_2_SO_4_, and 0.1 g/L FeCl_3_. Induction when DO picks up. The blue squares designate the concentrations of PCB, the orange circle represents the values of OD_600_, and the green triangle stands for the remaining amount of glycerol. E: The strains St26 and St07 fermented in GMD-Gly medium to 28 h in shake flasks. F: The strain St26 fermented in GMD-Gly fermentation medium to 36 h in 5-L bioreactor.

The relevant conditions for the fermentation in shake flasks were optimized for the best PCB biosynthetic strain, St26. This provided the basis for the subsequent expansion in a 5-L bioreactor. First, four fermentation mediums (TB, GMD, FLB, M9) were compared for the synthesis of PCB (Fig. 5A). Moreover, different induction temperatures were also compared for their effects on PCB synthesis (Fig. 5C). The optimal temperature for subsequent fermentation was 25 °C, which resulted in a titer of 32.2 mg/L. Because the main carbon source in the fermentation medium GMD-Gly was glycerol, the gene encoding glycerol kinase (*glpK*) of the strain St19 was mutated to improve the glycerol utilization [30]. Then, the plasmid PW26 was introduced into the strain St29, and the strain St30 was obtained. However, this result showed that mutations in the *glpK* gene lead to a decrease in PCB production (Fig. 5B). This may be caused by that mutated in gene *glpK* mainly targets downstream products that directly utilize glucose or glycerol metabolism.

Fed-batch cultivation of the strain St26 was carried out in a 5-L bioreactor using the GMD-Gly fermentation medium. To further increase PCB synthesis, the flow addition of MgSO_4_·7H_2_O, FeCl_3_, (NH_4_)_2_SO_4_, and glycerol was added, and the IPTG was added to induce PCB synthesis when the DO was increased to 40. The titer of PCB increased to 147.0 mg/L (Fig. 5D). Meanwhile, fed-batch fermentation was carried out using DO-stat. The DO was controlled at 40 by the *cis*-control method, with an aeration rate of approximately 4 vvm in the pre-fermentation period and 3 vvm after induction.

## 4 Discussion

In this study, suitable chassis strains for PCB synthesis were screened, and then two sources of key enzymes for PCB synthesis were optimized. The influence of different copy numbers on PCB synthesis was compared. Some key genes related to heme synthesis were integrated into the genome of the chassis strain. The genes negatively affecting heme synthesis were knocked-out to improve precursor supply, resulting in a PCB titer of 21.4 mg/L. We constructed HO1 and PcyA as a multi-enzyme complex via the RIAD-RIDD short peptide tag. In addition, the effects of different fermentation media on PCB synthesis were compared. We optimized the concentration and the addition time of the inducer during PCB fermentation in a 250-mL shake flask. In the GMD-Gly fermentation medium, 0.5 mM IPTG was added when OD_600_ = 0.6–1 to induce at 25 °C, and the PCB titer produced by strain St26 was 32.2 mg/L. Finally, the fermentation conditions of PCB were optimized in a 5-L bioreactor, including feed composition, dissolved oxygen, and rotational speed, to obtain PCB with the highest titer, 147.0 mg/L.

In the process of biosynthesis, precursor supply was the key factor to improve the titer of follow-up target products. During metabolic engineering to modify target strains for the synthesis of useful biobased chemicals, the synthesis of the target products requires a large supply of precursors [31,32]. For example, to enhance the synthesis of nicotinamide mononucleotide (NMN), the researchers directly added nicotinamide to the fermentation media and also enhanced the synthesis of 5-phosphoribosyll-pyrophosphate (PRPP), which ultimately promoted the synthesis of NMN [24]. During the synthesis of O-acetylhomoserine (OAH), the researchers enhanced the synthesis of its precursor substance l-homoserine with acetyl-CoA, resulting in a large accumulation of OAH [33]. Heme is a precursor of PCB, so an efficient supply of heme will likely promote the synthesis of PCB. Efficient synthesis of heme has been achieved in *E. coli* in previous studies [11], but the use of a large number of plasmids not only caused a large burden on the strain, but also consumed a large number of available plasmids [17,19,34]. Therefore, it was crucial to use an alternative means of enhancing the synthesis of the precursor heme. Integrating heterologous DNA into the genome enables stable expression and addresses the burden of plasmids, and researchers have listed several integration sites with stable expression through multiple assessments [34]. Thus, to minimize the cellular burden, the expression cassette for heme was integrated into the genome of *E. coli* BL21(DE3) at two locus, forming the final strain *E. coli* BL21(DE3) Δ*arsB*:*hemBCD* Δ*yfeX*:*hemEFGH* (St19). Finally, heme was supplied in significant levels, and this promoted the synthesis of PCB. This is a relatively common mode of conversion, and it is also a very efficient one.

High substrate concentrations have a substantial effect on the catalytic efficiency of enzymes. Some organelles in eukaryotes can accumulate large amounts of substrates, promoting the catalytic efficiency of specific enzymes. However, there are no organelles in prokaryotes, resulting in substrates being dispersed in the cytoplasm. Therefore, many researchers have tried to enhance the substrate concentration near the enzyme to promote the conversion rate of the target reaction. In a multi-enzyme reaction, the product synthesized by the previous enzyme is the substrate of the latter enzyme. By bringing the two enzymes closer to each other, the product released by the previous one can be directly captured by the latter enzyme. If two enzymes are connected by a linker, the target product can be efficiently synthesized [35, 36]. Furthermore, the efficiency of reactions that require more than two enzymes was improved by the DNA scaffold platform [[19], [37], [38]]. The construction of multi-enzyme complexes can control the flux of metabolites and reduce the diffusion of intermediates [[39], [40], [41]]. Similarly, we assembled the complex of Ho1 and PcyA using the two short peptide tags RIAD-RIDD to reduce BV IXα synthesis and promote heme to PCB synthesis.

In this study, the initial strain for PCB synthesis was constructed by screening the chassis strains, the source of the key enzymes, and the copy numbers. Most importantly, precursor supply was increased by genomic integration of key genes, which increased PCB titer by 10.5 mg/L compared with the initial strain. Furthermore, the key genes were self-assembled through the short peptide tags RIAD-RIDD to further increase PCB synthesis. The best fermentation medium was determined in a 250-ml shake flask. We optimized the feed composition, dissolved oxygen, and induction time in a 5-L bioreactor, which afforded a yield of 147.0 mg/L, the highest titer reported so far. However, there are still some unsolved problems in our research. The gene *hemAL* of the C5 pathway was also integrated, but unexpectedly, PCB was not even detected. We speculate that the use of the T7 promoter placed a significant burden on the chassis strain, resulting in a growth inhibition affecting subsequent synthesis, which is still being explored. In addition, we have not fully optimized Ho1 and PcyA, the two key enzymes for PCB synthesis. We plan to use enzyme engineering to systematically and rationally modify these enzymes to further improve the synthesis of PCB.

## Funding

This work was supported by the 10.13039/501100012166National Key Research and Development Program of China (2019YFA0904800) and the Foundation for Innovative Research Groups of the 10.13039/501100001809National Natural Science Foundation of China (32021005).

## CRediT authorship contribution statement

**Yuqi Wang:** Methodology, Investigation, Formal analysis, Writing – original draft, Writing – review & editing. **Ning Li:** Formal analysis, Writing – review & editing. **Xiaoyu Shan:** Methodology, Supervision. **Xinrui Zhao:** Investigation, Formal analysis. **Yang Sun:** Methodology, Supervision. **Jingwen Zhou:** Methodology, Supervision, Funding acquisition, Writing – review & editing.

## Declaration of competing interest

The authors declare that they have no known competing financial interests or personal relationships that could have appeared to influence the work reported in this paper.
