# Supplementary material for: Enhancement of phycocyanobilin biosynthesis in Escherichia coli by strengthening the supply of precursor and artificially self-assembly complex
Source: Synth Syst Biotechnol. 2023 Feb 28;8(2):227–34. doi: 10.1016/j.synbio.2023.02.005 (PMC10020671; doi:10.1016/j.synbio.2023.02.005)
Supplement: Multimedia component 1 [file mmc1.docx]

# Enhancement of phycocyanobilin biosynthesis in *Escherichia coli* by strengthening the supply of precursor and multienzyme complex

Yuqi Wang^1,3^, Ning Li^2,3^, Xiaoyu Shan^2,3^, Xinrui Zhao^2,3^, Yang Sun^1*^, Jingwen Zhou^1,2,3,4*^

^1^ Key Laboratory of Straw Comprehensive Utilization and Black Soil Conservation, Ministry of Education, College of Life Sciences, Jilin Agricultural University, Changchun, 130118, China;

^2^ Key Laboratory of Industrial Biotechnology, Ministry of Education and School of Biotechnology, Jiangnan University, 1800 Lihu Road, Wuxi, Jiangsu 214122, China;

^3^ Science Center for Future Foods, Jiangnan University, 1800 Lihu Road, Wuxi, Jiangsu 214122, China;

^4^ Jiangsu Provisional Research Center for Bioactive Product Processing Technology, Jiangnan University, 1800 Lihu Road, Wuxi, Jiangsu 214122, China.

* Corresponding authors: Yang Sun, Jingwen Zhou

Mailing address: College of Life Science, Key Laboratory of Straw Biology and Utilization, The Ministry of Education, Jilin Agricultural University, Changchun 130118, PR China

School of Biotechnology, Jiangnan University, 1800 Lihu Road, Wuxi, Jiangsu 214122, China

Phone: +86-510-85914371, Fax: +86-510-85914371

E-mail: ysun@jlau.edu.cn, zhoujw1982@jiangnan.edu.cn.

# Supporting Information

### Table S1 The strains applied and constructed in this study

| **Strain/plasmid** | **Description** | **Reference** |
| --- | --- | --- |
| *E. coli* BL21(DE3) | Used as host strain | Lab stock |
| *E. coli* JM109 | Used as cloning strain | Lab stock |
| St01 | *E. coli* Origami B(DE3) harboring pRSFDuet-T7lac-*ho1*^T^-T7lac-*pcyA*^S^ plasmids | This work |
| St02 | *E. coli* JM109(DE3) harboring pRSFDuet-T7lac-*ho1*^T^-T7lac-*pcyA*^S^ plasmids | This work |
| St03 | *E. coli* B834(DE3) harboring pRSFDuet-T7lac-*ho1*^T^-T7lac-*pcyA*^S^ plasmids | This work |
| St04 | *E. coli* W3110(DE3) harboring pRSFDuet-T7lac-*ho1*^T^-T7lac-*pcyA*^S^ plasmids | This work |
| St05 | *E. coli* BL21 Star(DE3) harboring pRSFDuet-T7lac-*ho1*^T^-T7lac-*pcyA*^S^ plasmids | This work |
| St06 | *E. coli* BL21(DE3) harboring pRSFDuet-T7lac-*ho1*^T^-T7lac-*pcyA*^S^ plasmids | Previous work^18^ |
| St07 | *E. coli* BL21(DE3) harboring pRSFDuet-T7lac-*ho1*^S^-T7lac-*pcyA*^S^ plasmids | This work |
| St08 | *E. coli* BL21(DE3) harboring pRSFDuet-T7lac-*ho2*^S^-T7lac-*pcyA*^S^ plasmids | This work |
| St09 | *E. coli* BL21(DE3) harboring pRSFDuet-T7lac-*ho1*^S^-*ho2*^S^-T7lac-*pcyA*^S^ plasmids | This work |
| St10 | *E. coli* BL21(DE3) harboring pRSFDuet-T7lac-*ho1*^S^-T7lac-*pcyA*^T^ plasmids | This work |
| St11 | *E. coli* BL21(DE3) harboring pRSFDuet-T7lac-*ho1*^T^-T7lac-*pcyA*^T^ plasmids | This work |
| St12 | *E. coli* BL21(DE3) harboring pRSFDuet-T7lac-*ho2*^S^-T7lac-*pcyA*^T^ plasmids | This work |
| St13 | *E. coli* BL21(DE3) harboring pRSFDuet-T7lac-*ho1*^S^-*ho2*^S^-T7lac-*pcyA*^T^ plasmids | This work |
| St14 | *E. coli* BL21(DE3) harboring pETDuet-T7lac-*ho1*^S^-T7lac-*pcyA*^S^ plasmids | This work |
| St15 | *E. coli* BL21(DE3) harboring pCDuet-T7lac-*ho1*^S^-T7lac-*pcyA*^S^ plasmids | This work |
| St16 | *E. coli* BL21(DE3) harboring pACYCDuet-T7lac-*ho1*^S^-T7lac-*pcyA*^S^ plasmids | This work |
| St17 | *E. coli* BL21(DE3) Δ*arsB*::*hemBCD* | This work |
| St18 | *E. coli* BL21(DE3) Δ*arsB*::*hemBCD* harboring pRSFDuet-T7lac-*ho1*^S^-T7lac-*pcyA*^S^ plasmids | This work |
| St19 | *E. coli* BL21(DE3) Δ*arsB*::*hemBCD* Δ*yfeX*::*hemEFGH* | This work |
| St20 | *E. coli* BL21(DE3) Δ*arsB*::*hemBCD* Δ*yfeX*::*hemEFGH* harboring pRSFDuet-T7lac-*ho1*^S^-T7lac-*pcyA*^S^ plasmids | This work |
| St21 | *E. coli* BL21(DE3) Δ*arsB*::*hemBCD* Δ*yfeX*::*hemEFGH* Δ*CysG* | This work |
| St22 | *E. coli* BL21(DE3) Δ*arsB*::*hemBCD* Δ*yfeX*::*hemEFGH* Δ*CysG*Δ*CyoE* | This work |
| St23 | *E. coli* BL21(DE3) Δ*arsB*::*hemBCD* Δ*yfeX*::*hemEFGH* Δ*CysG* harboring pRSFDuet-T7lac-*ho1*^S^-T7lac-*pcyA*^S^ plasmids | This work |
| St24 | *E. coli* BL21(DE3) Δ*arsB*::*hemBCD* Δ*yfeX*::*hemEFGH* Δ*CysG* Δ*CyoE* harboring pRSFDuet-T7lac-*ho1*^S^-T7lac-*pcyA*^S^ | This work |
| St25 | *E. coli* BL21(DE3) Δ*arsB*::*hemBCD* Δ*yfeX*::*hemEFGH* harboring pRSFDuet-T7lac-RIDD-GGGGS-*ho1*^S^-T7lac-RIAD-GGGGS-*pcyA*^S^ | This work |
| St26 | *E. coli* BL21(DE3) Δ*arsB*::*hemBCD* Δ*yfeX*::*hemEFGH* harboring pRSFDuet-T7lac-*ho1*^S^-GGGGS-RIDD-T7lac-RIAD-GGGGS-*pcyA*^S^ | This work |
| St27 | *E. coli* BL21(DE3) Δ*arsB*::*hemBCD* Δ*yfeX*::*hemEFGH* harboring pRSFDuet-T7lac-*ho1*^S^-GGGGS-RIDD-T7lac-*pcyA*^S^-GGGGS-RIAD | This work |
| St28 | *E. coli* BL21(DE3) Δ*arsB*::*hemBCD* Δ*yfeX*::*hemEFGH* harboring pRSFDuet-T7lac-RIDD-GGGGS-*ho1*^S^-T7lac-*pcyA*^S^-GGGGS-RIAD | This work |
| St29 | *E. coli* BL21(DE3) Δ*arsB*::*hemBCD* Δ*yfeX*::*hemEFGH* *glpK** | This work |
| St30 | *E. coli* BL21(DE3) Δ*arsB*::*hemBCD* Δ*yfeX*::*hemEFGH* *glpK** harboring pRSFDuet-T7lac-*ho1*^S^-GGGGS-RIDD-T7lac-RIAD-GGGGS-*pcyA*^S^ | This work |

### Table S2 Genes used in the present study

| Genes | Sequences |
| --- | --- |
| Ho1^S^ from *Synechocystis* sp. PCC6803 | ATGAGCGTTAACCTGGCGAGCCAGCTGCGTGAAGGCACCAAAAAATCTCACTCTATGGCGGAAAACGTTGGTTTCGTTAAATGCTTCCTGAAAGGTGTTGTTGAGAAAAACAGCTACCGTAAACTGGTTGGTAACCTGTACTTCGTTTACAGCGCGATGGAAGAAGAAATGGCGAAATTCAAAGATCACCCGATCCTGTCTCACATCTACTTCCCGGAACTGAACCGTAAACAGTCTCTGGAACAGGATCTGCAGTTCTACTATGGTAGCAACTGGCGTCAGGAAGTTAAAATCTCTGCGGCGGGTCAGGCGTACGTTGATCGCGTTCGTCAGGTTGCGGCGACCGCGCCGGAACTGCTGGTTGCGCACAGCTACACCCGTTACCTGGGCgcaCTGAGCGGCGGCCAGATCCTGAAGAAAATCGCGCAGAACGCGATGAACCTGCACGATGGTGGCACCGCGTTCTACGAATTCGCAGATATCGATGATGAAAAAGCGTTCAAAAACACCTACCGTCAGGCTATGAACGATCTGCCGATCGATCAGGCGACCGCGGAACGTATCGTTGATGAAGCGAACGATGCGTTCGCGATGAACATGAAAATGTTCAACGAACTGGAAGGTAACCTGATCAAAGCGATCGGCATCATGGTTTTCAACTCTCTGACCCGTCGTCGTAGCCAGGGCAGCACCGAAGTTGGTCTGGCGACCTCTGAAGGCTAA |
| Ho2^S^ from *Synechocystis* sp. PCC6803 | ATGACCAACCTGGCGCAGAAACTGCGCTATGGCACGCAGCAGAGCCATACCCTGGCGGAAAACACCGCGTATATGAAATGCTTTCTGAAAGGCATTGTGGAACGCGAACCGTTTCGTCAGCTGCTGGCGAACCTGTATTATCTGTATAGCGCGCTGGAAGCGGCGCTGCGTCAGCATCGCGATAACGAAATTATTAGCGCGATTTATTTTCCGGAACTGAACCGCACCGATAAACTGGCGGAAGATCTGACCTATTATTATGGCCCGAACTGGCAGCAGATTATTCAGCCGACCCCGTGCGCGAAAATTTATGTGGATCGCCTGAAAACCATTGCGGCGAGCGAACCGGAACTGCTGATTGCGCATTGCTATACCCGCTATCTGGGCGATCTGAGCGGCGGTCAGAGCCTGAAAAACATTATTCGCAGCGCGCTGCAGCTGCCGGAAGGCGAAGGCACCGCGATGTATGAATTTGATAGCCTGCCGACCCCGGGCGATCGCCGTCAGTTTAAAGAAATTTATCGCGATGTGCTGAACAGCCTGCCGCTGGATGAAGCGACCATTAACCGCATTGTGGAAGAAGCGAACTATGCGTTTAGCCTGAACCGCGAAGTGATGCATGATCTGGAAGATCTGATTAAAGCGGCGATTGGCGAACATACCTTTGATCTGCTGACCCGCCAAGATCGCCCGGGCAGCACCGAAGCGCGCAGCACCGCGGGCCATCCGATTACCCTGATGGTGGGCGAATAA |
| Ho1^T^ from *Thermosynechococcus* *elongatus*BP-1 | ATGACCACCAGCCTGGCGACCAAACTGCGTGAAGGCACCAAAAAAGCGCACACCATGGCGGAAAACGTTGGCTTCGTTCGTTGCTTCCTGAAAGGCACCGTTGAAAAAAGCTCTTACCGTAAACTGGTTGCGAGCCTGTACCACGTTTACAGCGCGATGGAACAGGAAATGGAACGTCTGAAAGATCACCCGATCGTTGGTAAAATCTACTTCCCGGAACTGAACCGTAAATCTTCTCTGGAACGTGATCTGACCTACTACTTCGGCTCTAACTGGCGTGAAGAAATCCCGCCGAGCCCGGCGACCCAGGCGTACGTTGCGCGTATCCACGAAGTTGCGAACACCGCGCCGGAACTGCTGGTTGCGCACAGCTACACCCGTTACCTGGGCGACCTGAGCGGCGGCCAGATCCTGAAAGGTATCGCGGAACGTGCGATGAACCTGCAGGATGGCGAAGGTACCGCGTTCTACCGTTTCGAATCCATCAGCGATGAAAAAGCGTTCAAACAGCTGTACCGTCAGCGTCTGGATGAACTGCCGGTTGATGAAGCGACCGCGGATCGTATCGTTGATGAAGCGAACGCGGCGTTCGGTATGAACATGAAAATCTTCCAGGAACTGGAAGGTAACCTGATCCGTGCGATCGGCCAGCTGCTGTTCAACACCCTGACCCGTCGTAAACAGCGTGGCAGCACCGAACTGGCGACCGCGGATTAA |
| PcyA^S^ from *Synechocystis* sp. PCC6803 | ATGGCGGTTACCGATCTGTCTCTGACCAACAGCAGCCTGATGCCGACCCTGAACCCGATGATCCAGCAGCTGGCGCTGGCGATCGCGGCGTCTTGGCAGAGCCTGCCGCTGAAACCGTACCAGCTGCCGGAAGATCTGGGTTACGTTGAAGGCCGTCTGGAAGGTGAAAAACTGGTTATCGAAAACCGTTGCTACCAGACCCCGCAGTTCCGTAAAATGCACCTGGAACTGGCAAAAGTGGGCAAAGGTCTGGATATCCTGCACTGCGTTATGTTCCCGGAACCGCTGTACGGCCTGCCGCTGTTCGGTTGCGATATCGTTGCGGGTCCGGGTGGTGTTAGCGCGGCTATCGCGGATCTGTCTCCGACCCAGTCTGATCGTCAGCTGCCGGCGGCGTACCAGAAATCTCTGGCGGAACTGGGTCAGCCGGAATTCGAACAGCAGCGTGAACTGCCGCCGTGGGGTGAAATCTTCTCTGAATACTGCCTGTTCATTCGTCCGTCTAACGTTACCGAAGAAGAACGTTTCGTTCAGCGTGTTGTTGATTTCCTGCAGATCCACTGCCACCAGAGCATCGTGGCGGAACCGCTGAGCGAAGCGCAGACCCTGGAACACCGTCAGGGCCAGATCCACTACTGCCAGCAGCAGCAGAAAAACGATAAAACCCGTCGTGTTCTGGAAAAAGCGTTCGGCGAAGCGTGGGCGGAACGTTACATGTCTCAGGTTCTGTTCGATGTTATCCAGTAA |
| PcyA^T^ from *Thermosynechococcus* *elongatus*BP-1 | ATGAGCCTGCGTCAGCACCAGCACCCGCTGATCCAGCGTCTGGCGGATCGTATCGAAGCGATTTGGCAGGCGTTCTTCCCGCTGGCGCCGTACGCGCTGCCGGAAGATCTGGGCTACGTTGAAGGCAAACTGGAAGGCGAACGTCTGACCATCGAAAACCACTGCTACCAGGCGCCGCCGTTCCGTAAACTGCACCTGGAACTGGCGCGTGTTGGCGAATCTCTGGATATCCTGCACTGCGTTATGTTCCCGGAACCGCGTTACGATCTGCCGATGTTCGGTTGCGATCTGGTTGGTGGTCGTGGCCAGATCAGCGCGGCGATCGTTGATCTGTCTCCGGTTACCGGCCAGCTGCCGGCGGCGTACACCTGCGCGCTGAACGCGCTGCCGAAACTGACCTTCCGTCAGCCGCGCGAACTGCCGCCGTGGGGTCACATCTTCAGCCCGTTCTGCATCTTCATCCGTCCGCAGGGCGAAGCGGAAGAACAGCAGTTCCTGGATCGCATCGGTGAATACCTGACCCTGCACTGCCAGCTGTCTCAGCAGGCGGTTCCGACCGATCACCCGCAGGCGGTTATCGCGGGTCAGCGTCAGTACTGCCAGCAGCAGCAGCAGAACGATAAAACCCGTCGCGTTCTGGAAAAAGCGTTCGGTGTTCCGTGGGCGGAACGTTACATGACCACCGTGCTGTTCGATGTTCCGCCGGTTTAA |
| HemB from *Escherichia coli* BL21(DE3) | acagacttaatccaacgccctcgtcgcctgcgcaaatctcctgcgctgcgcgctatgtttgaagagacaacacttagccttaacgacctggtgttgccgatctttgttgaagaagaaattgacgactacaaagccgttgaagccatgccaggtgtgatgcgcattccagagaaacatctggcacgcgaaattgaacgcatcgccaacgccggtattcgttccgtgatgactttcggcatctctcaccataccgatgaaaccggcagcgatgcctggcgggaagatggactggtggcgcgaatgtcgcgcatctgcaagcagaccgtgccagaaatgatcgtcatgtcagacacctgcttctgcgaatacacatctcacggtcactgcggtgtgctgtgcgagcatggcgtcgacaacgacgcgactctggaaaatttaggcaagcaagccgtggttgcagctgctgcaggcgcagacttcatcgccccttctgccgcgatggacggccaggtacaggcgattcgccaggcgctggacgctgcgggctttaaagatacggcgattatgtcgtattcgaccaagttcgcctcttccttttatggtccgttccgtgaagctgccggaagcgcattaaaaggcgaccgcaaaagctatcagatgaacccaatgaaccgtcgtgaggcgattcgtgagtcactgctggatgaagcccagggcgcagactgtctgatggttaaacctgccggagcgtacctcgacatcgtgcgtgagctgcgtgaacgtactgaattgccgattggcgcgtatcaggtgagcggtgagtacgcgatgattaagttcgccgcgctggcgggtgctatagatgaagagaaagtcgtgctcgaaagcttaggttcaattaagcgtgcgggtgcggatctgattttcagctactttgcgatggatttggctgagaagaagattctgcgttaa |
| HemC from *Escherichia coli* BL21(DE3) | ttagacaatgttttaagaattgccacacgccaaagcccacttgcactctggcaggcacactatgtcaaagacaagttgatggcgagccatccgggcctggtcgttgaactggtaccgatggtgacgcgcggcgatgtgattcttgatacgccgctggcgaaagtaggcggaaaaggcttatttgttaaagagctggaagtcgcgctcctcgaaaatcgcgccgatatcgccgtacattcaatgaaagatgtgccggttgaattcccgcaaggtctgggactggtcactatttgtgagcgtgaagatcctcgcgatgcctttgtgtccaataactatgacaatctggatgcgttaccggcaggcagtatcgtcgggacgtccagtttacgtcgccagtgccaactggctgaacgccgcccggatctgattatccgctccctgcgaggcaacgtcggcactcgcctgagtaaactggataacggcgaatacgatgccatcattcttgcggtagccggactaaaacgtttaggtctggagtcccgcattcgcgccgcattgccacccgagatttctcttccggcggtaggacaaggtgcggtgggtattgaatgccgccttgatgattctcgcactcgcgagctgcttgccgcgctgaatcaccacgaaactgcactgcgcgttaccgcagaacgcgccatgaatacccgtctcgaaggcggatgtcaggtgccaattggtagctacgccgagcttattgatggcgaaatctggctgcgtgcgttggtcggcgcgccggacggttcgcagattattcgcggtgaacgccgcggtgcgccgcaagatgccgaacaaatggggatttcgctggcagaagagctactgaataacggcgcgcgcgagatcctcgctgaagtctataacggagacgctccggcatga |
| HemD from *Escherichia coli* BL21(DE3) | atgagtatcctggtcacccgcccgtctcccgctggagaagagttagtgagccgtctgcgcacactggggcaggtggcctggcattttccactgattgagttttctccgggtcgacaattaccacaacttgctgatcaactggcggcgctgggggagagcgatctgttgtttgccctctcgcaacacgcggttgcttttgcccaatcacagctgcatcagcaagatcgtaaatggccccgactacctgattatttcgccattggacgcaccaccgcactggcactacataccgtaagcggacagaagattctctacccgcaggatcgggaaatcagcgaagtcttgctacaattacctgaattacaaaatattgcgggcaaacgtgcgctgatattacgtggcaatggcggtcgtgagctaattggggataccctgacggcgcgcggtgctgaggtcactttttgtgaatgttatcaacgatgcgcaatccattacgatggtgcagaagaagcgatgcgctggcaatcccgcgaggtgacgacggtcgttgttaccagcggtgaaatgttgcagcaactctggtcgctgatcccacaatggtatcgtgagcactggttactacactgtcgactattggtcgtcagtgagcgtttggcgaaactcgcccgggaactgggctggcaagacattaaggtcgccgataacgctgacaacgatgcgcttttacgggcattacaataa |
| HemE from *Escherichia coli* BL21(DE3) | accgaacttaaaaacgatcgttatctgcgggcgctgctgcgccagcccgttgatgtcactccagtatggatgatgcgccaggcgggtcgctatctaccggaatataaagccacgcgcgcccaggcgggcgattttatgtcgctgtgcaaaaacgccgagctggcgtgcgaagtgactttgcaaccgctgcgtcgctacccgctggatgcggcgatcctcttttccgatatcctcaccgtgccggacgcgatggggttagggctctattttgaagccggagaaggtccgcgttttacctcgccagtcacctgcaaagccgacgtcgataaactgccaattccggacccggaagatgagctgggttacgtgatgaacgcggtgcgtaccattcgtcgcgaactgaaaggcgaagtgccgctgattggtttttccggcagcccgtggacgctggcgacctacatggtggaaggcggcagcagcaaagcgttcaccgtgatcaaaaaaatgatgtatgccgatccgcaggcgctgcacgctctactcgataaactggcgaaaagcgtcactttgtatctgaatgcgcagattaaagccggtgctcaggcagtgatgattttcgacacctggggcggtgtgcttaccgggcgcgattatcaacagttctcgctctattacatgcataaaattgttgatggtttactgcgtgaaaacgacggtcgccgcgtaccggtcacgctgtttaccaaaggcggcggacagtggctggaagcgatggcagaaaccggttgcgatgcgttgggcctcgactggacaacggatatcgccgatgcgcgccgccgtgtgggcaataaagtcgcgttgcagggtaatatggatccgtcgatgctgtacgctccgcctgcccgcattgaagaagaagtagcgactatacttgcaggtttcggtcacggcgaaggtcatgtctttaaccttggtcacggcattcatcaggatgtgccgccagaacatgctggcgtattcgtggaggcagtgcatcgactgtctgaacaatatcaccgctaa |
| HemF from *Escherichia coli* BL21(DE3) | atgaaacccgacgcacaccaggttaaacagtttctgctcaaccttcaggatacgatttgtcagcagctgaccgccgtcgatggcgcagaatttgtcgaagatagttggcagcgcgaagctggcggcggcgggcgtagtcgggtgttgcgtaatggtggtgttttcgaacaggcaggcgtcaacttttcgcatgtccacggtgaggcgatgcctgcttccgccaccgctcatcgcccggaacttgccgggcgcagtttcgaggcgatgggcgtttcactggtagtgcatccgcataacccgtatgttcccaccagccacgcgaatgtgcggttttttattgccgaaaaaccgggtgccgatcccgtctggtggtttggcggcggcttcgatttaacccctttctatggttttgaagaagacgccattcactggcaccgcaccgcccgtgacctgtgcctgccatttggtgaagacgtttatccccgttacaaaaagtggtgcgacgattacttctacctcaaacatcgcaacgaacagcgcggtattggcgggctgttctttgatgatctgaacacgccagatttcgaccactgttttgcctttatgcaggcggtaggcaaaggctacaccgacgcttatttaccaattgtagagcgacgtaaagcgatggcctacggcgagcgcgagcgcaattttcagctctaccgtcgcggtcgttatgtcgagttcaatctggtctgggatcgcggcacgctgtttggcctgcaaactggcgggcgcaccgagtctatcctgatgtcaatgccgccactggtacgctgggaatatgattatcagccaaaagatggcagcccagaagcggcgttaagtgagtttattaaggtcagggattgggtgtaa |
| HemG from *Escherichia coli* BL21(DE3) | gtgaaaacattaattcttttctcaacaagggacggacaaacgcgcgagattgcctcctacctggcttcggaactgaaagaactggggatccaggcggatgtcgccaatgtgcaccgcattgaagaaccacagtgggaaaactatgaccgtgtggtcattggtgcttctattcgctatggtcactaccattcagcgttccaggaatttgtcaaaaaacatgcgacgcggctgaattcgatgccgagcgccttttactccgtgaatctggtggcgcgcaaaccggagaagcgtactccacagaccaacagctacgcgcgcaagtttctgatgaactcgcaatggcgtcccgatcgctgcgcggtcattgccggggcgctgcgttacccacgttatcgctggtacgaccgttttatgatcaagctgattatgaagatgtcaggcggtgaaacggatacgcgcaaagaagttgtctataccgattgggagcaggtggcgaatttcgcccgagaaatcgcccatttaaccgacaaaccgacgctgaaataa |
| HemH from *Escherichia coli* BL21(DE3) | cgtcagactaaaaccggtatcctgctggcaaacctgggtacgcccgatgcccccacacctgaagcggtaaaacgctatctgaaacaatttttaagcgacagacgcgtggttgatacctcacggttgttatggtggccattgctgcgcggcgtgattttgccgctgcgctcgccgcgtgtggcgaagctgtatgcctctgtctggatggaaggtggctcgccgctgatggtttacagccgtcagcaacagcaggcgctggcacaacgtttaccggagacgcccgtagcgctgggaatgagctacggctcgccatcactggaaagcgccgtagatgaactcctggcagagcatgtagatcatattgtggtgctgccgctttatccgcaatactcctgttcaacggtcggtgcggtatgggatgaactggcacgcattctggcgcgcaaacgtagcattccggggatatcgtttattcgtgattacgctgataaccacgattacattaatgcactggcgaacagcgtacgcgcttcttttgccaaacatggcgaaccggatctgctgctgctctcttatcatggcattccccagcgttatgcagatgaaggcgatgattacccgcaacgttgccgcacaacgactcgcgaactggcttccgcactggggatggcaccggaaaaagtgatgatgacctttcagtcgcgctttggtcgggaaccctggctgatgccttataccgacgaaacgctgaaaatgctcggagaaaaaggcgtaggtcatatacaggtgatgtgcccgggctttgctgcggattgtctggagacgctggaagagattgccgagcaaaaccgtgaggtcttcctcggtgccggcgggaaaaaatatgaatatattccagcgcttaatgccacgccggaacatattgaaatgatggctaatcttgttgccgcgtatcgctaa |
| RIDD | GGTGGTGGTGGTTCAGGTGGTGGTGGTTCAGGTGGTGGTGGTTGTGGTAGCCTGC GTGAATGTGAACTGTATGTTCAGAAACATAATATTCAGGCCCTGCTGAAAGATAGC ATTGTTCAGCTGTGTACCGCACGTCCGGAACGTCCGATGGCATTTCTGCGCGAATA TTTTGAACGTCTGGAAAAAGAAGAAGCCAAA |
| RIAD | GGTGGTGGTGGTTCAGGTGGTGGTGGTTCAGGTGGTGGTGGTTGTGGTCTGGAACAGTATGCAAATCAGCTGGCAGATCAGATTATCAAAGAAGCAACCGAAGGTTGC |

### Table S3 Primers used in the pathway construction

| **Primers** | **Sequences** |
| --- | --- |
| HemBCD-F | TAATACGACTCACTATAGGGGAATTGTGAGC |
| HemBCD-R | CTAAACGGGTCTTGAGGGGTTTTTTG |
| HemEFGH-F | GATCTCGACGCTCTCCCTTATGC |
| HemEFGH-R | TCTAAACGGGTCTTGAGGGGTTTTTTG |
| UP-arsB-F | CTATTACCTTCCTCTGCACTTACACATTCG |
| UP-arsB-R | TGAGATACTGATATGAGCAACATTACCATTTATCACAAC |
| DN-arsB-F | GATGTGTTTTATCCGGGAGGCATT |
| DN-arsB-R | GTTATTTGTAGGCCTGACAGGCGTAGCGCATCAGGC |
| UP-yfeX-F | TGTTCCTCCTGAAAATAAGAATGCCACG |
| UP-yfeX-R | CACTTTGGTGGACGACCCAAAC |
| DN-yfeX-F | GTGTATTACGCAGTCAGGTACGC |
| DN-yfeX-R | TGGCCTTTAATCAATGAATCAGAAACGC |
| UP-CysG-F | GCAGCATCAGCCACGGG |
| UP-CysG-R | ACGGCTGCCGGTTAATTACTAAGGGGTTTTTAC |
| DN-CysG-F | TTTAACCCGGGCCAGAGAATTCTGGCC |
| DN-CysG-R | GCGTATTTGTTTCTATGTCCTTCTTGCCC |
| UP-CyoE-F | CCGCAATCAAACTGTTTTTGCCC |
| UP-CyoE-R | CCGTAGCACCTTTTTAATAGAGAGGTTTTG |
| DN-CyoE-F | AACCGCCGCTCTTAGTGC |
| DN-CyoE-R | CGCCATCCAGATAAGACCGGAAG |
| gRNA-arsB-F | GAAGATGACAATCTGCCAGGGTTTTAGAGCTAGAAATAGCAAGTTAAAATAAG |
| gRNA-arsB-R | CTCTAAAACCCTGGCAGATTGTCATCTTCACTAGTATTATACCTAGGACTGAGCTAG |
| gRNA-yfeX-F | GATCAACACATCGAACTGGGGTTTTAGAGCTAGAAATAGCAAGTTAAAATAAG |
| gRNA-yfeX-R | CTCTAAAACCCCAGTTCGATGTGTTGATCACTAGTATTATACCTAGGACTGAGCTAG |
| gRNA-CysG-F | TGCTGGAAACACTGTGCAACGGTTTTAGAGCTAGAAATAGCAAGTTAAAATAAGGCTAG |
| gRNA-CysG-R | CTAAAACCGTTGCACAGTGTTTCCAGCACTAGTATTATACCTAGGACTGAGCTAGCTG |
| gRNA-CyoE-F | TCAACGGACATCATCACCGAGGTTTTAGAGCTAGAAATAGCAAGTTAAAATAAGGCTAG |
| gRNA-CyoE-R | CTAAAACCTCGGTGATGATGTCCGTTGACTAGTATTATACCTAGGACTGAGCTAGCTG |


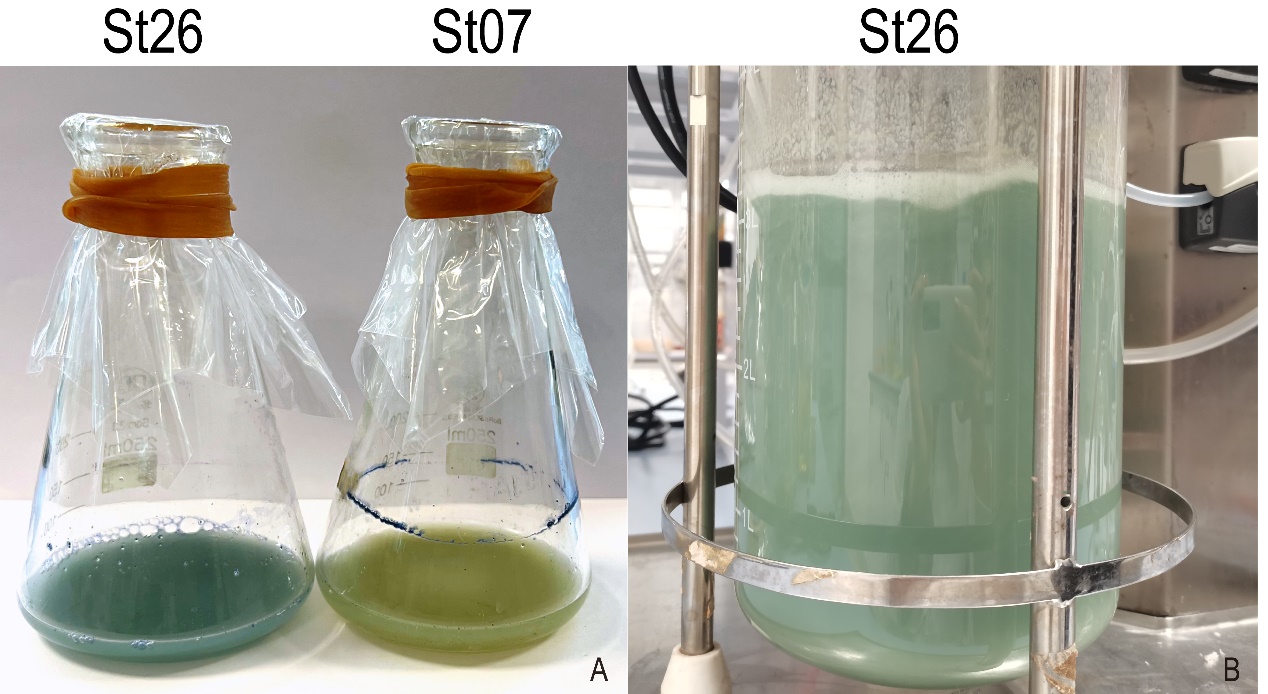


**Figure S1 The fermentation color of different strains.** A: The strains St26 and St07 fermented in GMD1 medium to 28 h in shake flasks; B: The strains St26 fermented in GMD1 medium to 36 h in 5-L bioreactor.


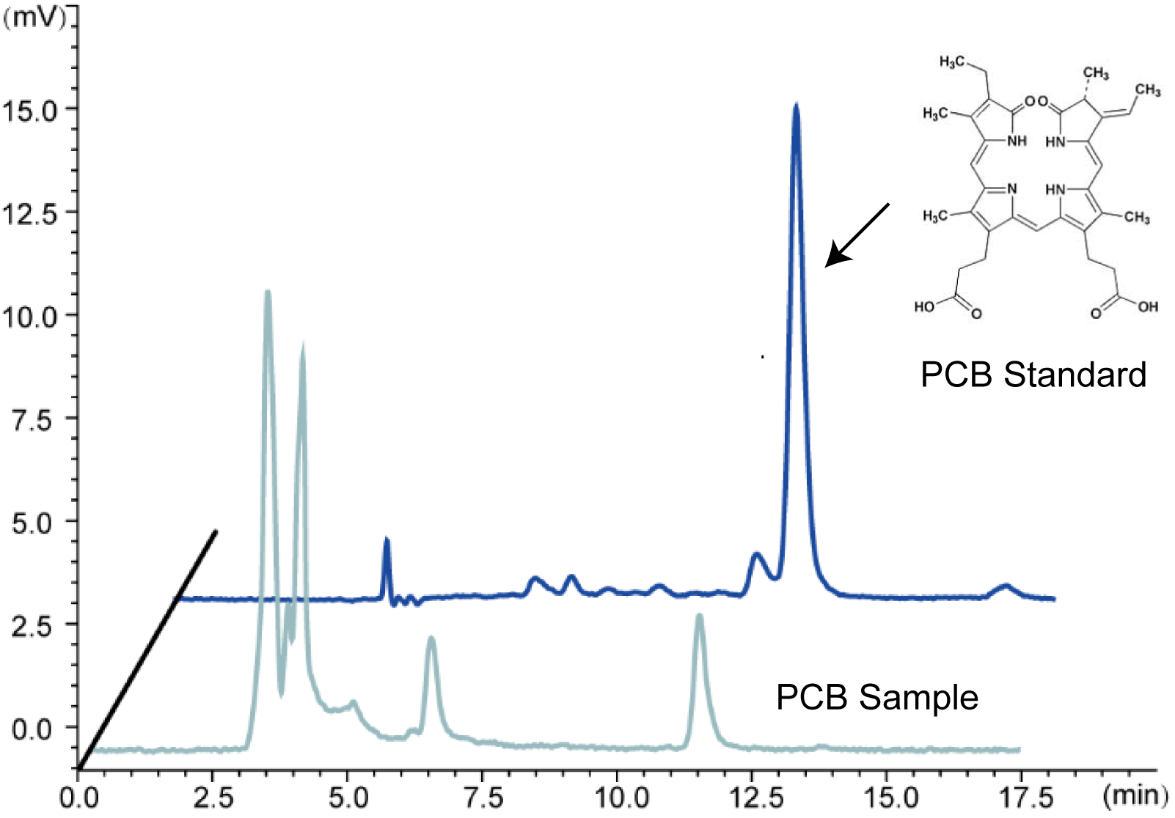


**Figure S2 HPLC map of the PCB sample St07 and PCB standard.**
